# Dyskinesia-Hyperpyrexia Syndrome in Parkinson’s Disease May Benefit from GPi Deep Brain Stimulation: A Case Report

**DOI:** 10.5334/tohm.912

**Published:** 2025-05-07

**Authors:** Shi-Shi Huang, Lu-Lu Ma, Han-Song Sheng, Rong-Pei Liu, Shi-Guo Zhu, Shan-Shan Xu, Mei-Ling Sheng, Zhu-Ling Chen, Nu Zhang, Xiong Zhang, Jian-Yong Wang

**Affiliations:** 1 Department of Neurology, Institute of Geriatric Neurology, the Second Affiliated Hospital and Yuying Children’s Hospital, Wenzhou Medical University, Wenzhou, Zhejiang, China; 2 Department of Neurosurgery, the Second Affiliated Hospital and Yuying Children’s Hospital, Wenzhou Medical University, Wenzhou, Zhejiang, China

**Keywords:** Parkinson’s disease, Dyskinesia-hyperpyrexia syndrome, Deep brain stimulation, Globus pallidus internus, Dyskinesia

## Abstract

**Background:**

Dyskinesia-hyperpyrexia syndrome (DHS) is a rare but life-threatening complication in Parkinson’s disease (PD). Its prevention and treatment are clinical challenges.

**Case report:**

We herein present a case of a female PD patient who experienced recurrent episodes of DHS and levodopa-induced dyskinesia. The complications were alleviated after bilateral globus pallidus internus (GPi) deep brain stimulation (DBS).

**Discussion:**

Our report provides evidence that GPi DBS may reduce the susceptibility to DHS. More evidences are warranted to validate this hypothesis.

## Introduction

In advanced stage of Parkinson’s disease (PD), motor complications including dyskinesia greatly reduce the quality of life in PD patients and limit the use of antiparkinsonian drugs [1]. Sometimes, unexpected emergencies such as dyskinesia-hyperpyrexia syndrome (DHS) and parkinsonism-hyperpyrexia syndrome (PHS) may threaten them [2].

DHS is a rare but critical complication in PD, which was first defined in 2010 [3]. Similar but different from PHS, DHS is characterized by hyperthermia, continuous dyskinesia, altered mental status, and elevated creatinine kinase (CK). Based on the reported cases, DHS has a mortality rate of 15.4%. The pathogenesis of DHS remains unclear, though a number of factors including antiparkinsonian drug abuse/change, infection, heatwave, gastrointestinal dysmotility, and trauma have been reported as potential triggers [4].

The remission of DHS mainly relies on treatment of triggers, reduction of dopaminergic drugs, vital function support, and antipyretic measures [4]. DHS may recur in some PD patients, and it seems difficult to prevent the episodes [5,6].

Herein, we reported a female PD patient who experienced recurrent episodes of DHS, which was followed by levodopa-induced dyskinesia (LID). Her symptoms improved dramatically after bilateral globus pallidus internus (GPi) deep brain stimulation (DBS).

## Case presentation

The patient was a 72-year-old woman with a 15-year history of PD. Her medical history included hypertension and diabetes. She was treated with benserazide/levodopa 12.5/50 mg four times daily, selegiline 5 mg daily, levodopa/carbidopa/entacapone 100/50/200 mg twice daily, and amantadine 100 mg twice daily. She has regular follow-up visits, and she did not suffer any motor fluctuation.

In her 15th year of suffering from PD, the patient was transported to the emergency center of a hospital for severe confusion, high fever and dyskinesia during the last 24 hours. She was diagnosed with DHS, and the intensive care unit treatment was initiated. The bacteriological culture of blood was sterile and the computerized tomography (CT) scan of chest was negative. The treatment included vital function support, intravenous fluids, broad-spectrum antibiotics, and antipyretic measures. After half a month of treatment, the patient was recovered. Her dopaminergic medication remained unchanged after she was discharged from the hospital. Since then, she developed mild dyskinesia.

One month later, she visited neurology department of our hospital for hyperthermia, severe dyskinesia, confusion and lethargy in the past 6 days. On admission, she was in a confusional state. Her temperature was 38.5°C, and her heart rate was 108 beats per minute. Severe incessant dyskinesia was observed in head, neck, trunk and limbs (Supplementary Videos 1). No meningeal signs were detected. Blood tests showed high levels of CK (610 IU/L) and myohemoglobin (72 ng/mL). Her chest CT scan, cranial magnetic resonance imaging, and blood bacterial cultures were negative.

Based on the continuous generalized dyskinesia, hyperthermia, altered mental status, and elevated CK level, we diagnosed the patient as DHS, and suspended her antiparkinsonian medication. Intravenous midazolam and normal saline were administered for sedation and hydration. At the same time, physical antipyretic measures were administered to lower her body temperature. Her clinical condition gradually improved. On the third day, her body temperature returned to a normal level, and her dyskinesia disappeared completely. The CK and myohemoglobin levels became normal within one week. Meanwhile, her parkinsonism symptoms re-emerged (Supplementary Videos 2), so we prescribed her with a low dose of levodopa/carbidopa/entacapone (50/25/100 mg three times daily). However, the patient developed dyskinesia over four limbs, trunk, neck, and tongue, but her rigidity and bradykinesia were not well relieved (Supplementary Videos 3). Her Movement Disorder Society Unified Parkinson’s Disease Rating Scale (MDS-UPDRS) total score was 113, with 59 points in motor examination (part III) and 16 points in motor complications (part IV). After reaching an agreement with the patient and her family, we performed DBS treatment system implantation of bilateral GPi, which site is more advantageous in reducing dyskinesia [7]. One month later, bilateral electrodes began to stimulate with amplitude at 4.3 V on both sides, pulse width at 90 µs on the left and 110 µs on the right, and frequency at 155 Hz bilaterally. During the follow-up for more than one year, the patient’s symptoms improved significantly and she did not suffer DHS and dyskinesia, even though her antiparkinsonian medication was only benserazide/levodopa 12.5/50 mg three times daily (Supplementary Videos 4). The patient was satisfied with the treatment.

## Discussion

DHS is a rare but life-threatening complication in PD. At the same time, LID is of great concern to PD patients and physicians, because it limits the use of drugs and greatly reduces the quality of life in patients. In our report, the PD patient experienced recurrent DHS and LID. The complications were greatly alleviated after bilateral GPi DBS.

The underlying mechanism of DHS is poorly understood. It has been proposed that massive dyskinetic movements may lead to hyperthermia and rhabdomyolysis, which further exacerbate the confusional state [3]. It is also believed that autonomic dysfunction, impaired thermoregulation, and reduced dopamine buffering capacity of the striatum contribute to the development of DHS [5,8].

Interestingly, like two other previously reported cases [5,6], our PD patient experienced recurrent episodes of DHS. It seems that certain populations are more susceptible to developing DHS. Previous research showed that hypothalamic dopamine release is involved in thermoregulation [9], while dyskinesia is more related to the dopamine pathway in the striatum [10]. Here, we propose that in some specific PD patients, in addition to abnormalities in the nigrostriatal pathway, the hypothalamic thermoregulatory pathway is also impaired. Triggers such as overusing of antiparkinsonian drug and heatwave contributed to the deterioration of both pathways, which further led to the onset of DHS.

Notably, after the episodes of DHS, the patient developed LID which was induced by low dose dopaminergic drug. This phenomenon has not been reported before. It is believed that dyskinesia is associated with abnormal signaling between motor cortex and striatum, which was resulted from the combination of nigral denervation and chronic use of antiparkinsonian drugs [11]. What we observed in this patient suggests that DHS may impair related signaling pathways, making it more likely to develop LID.

Treatment of DHS mainly involves vital function support, reduction of antiparkinsonian drugs, intravenous fluids, and antipyretic measures. The underlying triggers should be avoided and treated immediately [4]. Our patient experienced 2 episodes of DHS successively in July and August. Since the patient did not take any drugs for overdose, we believe that high ambient temperature may be its precipitating factor, as has been reported in previous cases [5]. After the bilateral GPi DBS treatment, she was relieved from DHS episodes and dyskinesia. This improvement may come from the reduction of dopaminergic drugs, and it may also be related to GPi stimulation. Furthermore, although previous studies showed that stimulation in GPi is not as effective as subthalamic nucleus in reducing medication [12], we found that GPi stimulation does have a certain effect in reducing medication in this patient.

The mechanism of DBS remains unclear. It has been proposed that the pathological brain circuit activity of PD may be disrupted by stimulation at ionic, protein, cellular and network levels [13]. DBS is effective in improving motor symptoms in patients with advanced PD, and GPi DBS is especially effective for dyskinesia [14]. Whether it has an effect on reducing susceptibility to DHS requires more observation.

In summary, we herein present a case of PD patient who experienced recurrent DHS and LID. The complications were alleviated after bilateral GPi DBS. We suggest that DHS may benefit from GPi DBS. Its long-term efficacy requires longer observation. More reports are warranted to validate this hypothesis.

## Additional File

The additional file for this article can be found as follows:

10.5334/tohm.912.s1 Supplementary videos. Supplementary videos 1 to 4.
