# Supplementary material for: Dyskinesia-Hyperpyrexia Syndrome in Parkinson’s Disease May Benefit from GPi Deep Brain Stimulation: A Case Report
Source: Tremor Other Hyperkinet Mov (N Y). 2025 May 7;15:20. doi: 10.5334/tohm.912 (PMC12063572; doi:10.5334/tohm.912)
Supplement: Supplementary videos. — Supplementary videos 1 to 4. [file tohm-15-1-912-s1.zip › tohm-15-1-912-s1/67127ee6b03f8.docx]

**Supplementary Video legend**

**Supplementary Video 1:** Continuous dyskinesia was observed over her head, trunk, and four limbs. One day after last dose of levodopa.

**Supplementary Video 2:** Parkinsonism symptoms re-emerged.

**Supplementary Video 3:** After taking a low dose of levodopa/carbidopa/entacapone, the patient developed dyskinesia over four limbs, trunk, neck, and tongue, but her rigidity and bradykinesia were not well relieved.

**Supplementary Video 4:** After bilateral electrodes began to stimulate, the patient’s parkinsonism symptoms improved without suffer any dyskinesia. The video was recorded in the “off” medication state.
